# Allergic Bronchopulmonary Aspergillosis

**DOI:** 10.3390/jof2020017

**Published:** 2016-06-06

**Authors:** Michael C. Tracy, Caroline U. A. Okorie, Elizabeth A. Foley, Richard B. Moss

**Affiliations:** Center for Excellence in Pulmonary Biology, Department of Pediatrics, Stanford University School of Medicine, 770 Welch Road suite 350, Palo Alto, CA 94304, USA; mtracy@stanford.edu (M.C.T.); cokorie@stanford.edu (C.U.A.O.); lizfoley@stanford.edu (E.A.F.)

**Keywords:** asthma, cystic fibrosis, *Aspergillus fumigatus*

## Abstract

Allergic bronchopulmonary aspergillosis (ABPA), a progressive fungal allergic lung disease, is a common complication of asthma or cystic fibrosis. Although ABPA has been recognized since the 1950s, recent research has underscored the importance of Th2 immune deviation and granulocyte activation in its pathogenesis. There is also strong evidence of widespread under-diagnosis due to the complexity and lack of standardization of diagnostic criteria. Treatment has long focused on downregulation of the inflammatory response with prolonged courses of oral glucocorticosteroids, but more recently concerns with steroid toxicity and availability of new treatment modalities has led to trials of oral azoles, inhaled amphotericin, pulse intravenous steroids, and subcutaneously-injected anti-IgE monoclonal antibody omalizumab, all of which show evidence of efficacy and reduced toxicity.

## 1. Introduction

In the spectrum of disease caused by *Aspergillus* species, in particular *Aspergillus fumigatus*, by far the greatest number of affected individuals, predisposed by underlying asthma or cystic fibrosis, suffer from allergic respiratory manifestations [1,2]. Lung disease resulting from exposure to *Aspergillus* and maladaptive immune responses span a spectrum of phenotypic severity, from exacerbation of simple fungal asthma to severe asthma with fungal sensitization to allergic bronchopulmonary aspergillosis (ABPA) [3,4,5]. In this review we will consider recent advances in understanding the underlying host response mechanisms responsible for the dichotomy between invasive and allergic disease due to *Aspergillus*, examine the evolution of diagnostic criteria and procedures, and summarize the expanding therapeutic options in managing ABPA. 

### 1.1. Pathogenesis of ABPA

**Exposure.** The fungal genus *Aspergillus* is ubiquitous in the environment and, thus, the inhalation of *Aspergillus* spores is unavoidable. *Aspergillus fumigatus* is an airborne filamentous saprophytic species that lives in soil, and is found commonly in compost and water-damaged structures. Given that *A. fumigatus* spores are 3–5 µm in size, they can readily deposit in the lower bronchial airways [6]. In a host with normal immunologic function, inhaled *Aspergillus* conidia are cleared from the airway without associated morbidity. However, *Aspergillus fumigatus* is a species that has a formidable array of virulence and immunoevasive properties contributing to its pathogenic potential that lead to its predominance in allergic, as well as invasive, fungal disease [7].

**Colonization.** Susceptible hosts include individuals with cystic fibrosis (CF) or asthma. Both of these populations have abnormalities in their airway mucosal defenses, including mucociliary clearance and epithelial cell function [8]. Exposure to elevated concentrations of *Aspergillus* conidia have been associated with cases of ABPA. However, there is wide variability in clinical response, as only a subset of patients develop sensitization to *Aspergillus.* A systematic review and meta-analysis of 21 studies in asthmatics reported a pooled prevalence of *Aspergillus* sensitization of 28%, and of 12.9% for ABPA [9]. With regard to CF, a similar meta-analysis of 64 studies revealed a pooled prevalence of *Aspergillus* sensitization of 39.1%, and of 8.9% for ABPA. This study further noted that adults had a slightly higher prevalence of ABPA (10.1%) than did children (8.9%) [10].

There appears to be a genetic predisposition to developing ABPA, which is supported by work showing a familial occurrence of 4.9% [11]. Both asthma and CF adversely affect mucociliary clearance, likely contributing to a reduced ability to rapidly clear inhaled *Aspergillus* conidia before contact of fungal elements with the innate immune system and, thereby, facilitating fungal growth and mucosal colonization. There are an increasing number of reports of mutations and polymorphisms in host response genes found in ABPA patients which suggest a panoply of underlying abnormalities in both adaptive and innate immunity [12]. Of note, heterozygous mutations in the cystic fibrosis transmembrane conductance regulator gene (the cause of cystic fibrosis when both alleles are mutated) appear to occur more commonly in patients with ABPA than in asthmatics or the general population [13]. On chromosome 6, alleles in the HLA class II region are associated with susceptibility to ABPA in CF as well as asthma [14,15]. Collectively, these genetic susceptibility factors likely contribute to the persistence of *Aspergillus* conidia, germination and hyphal growth in the airway, and/or abnormal immunoinflammatory responses.

**Immune response**. *Aspergillus* conidia cell walls are covered by a surface layer of rodlet proteins and melanin, which are hydrophobic and immunologically inert and, thus, do not provoke an inflammatory response [16]. However, in susceptible hosts, the conidia swell and germinate resulting in hyphal growth that leads to a robust inflammatory response. The immune system response to *A. fumigatus* swollen conidia and hyphae begins with the recognition of newly exposed pathogen-associated molecular patterns (PAMPs) by innate immune cells. PAMP constituents of the *A. fumigatus* cell wall include β-glucan, chitin, galactomannan, and galactosaminogalactan [17,18]. Innate immune cells recognize PAMPs through pattern recognition receptors (PRRs) present on epithelia and “professional” antigen presenting cells (APCs) such as dendritic cells. PRRs identified in invasive aspergillosis include C-type lectin receptors (dectin-1), Toll-like receptors (especially TLR2 and TLR4), and nucleotide-binding oligomerization domain-like receptors [19]. Activated PRRs trigger APCs, primarily dendritic cells, to release chemokines and cytokines, which culminate in adaptive immune T-helper cell responses [20]. Th1 activation is associated with an effective pro-inflammatory response characterized by macrophage and neutrophil phagocytosis and clearance of *Aspergillus* conidia [21].

Unlike invasive aspergillosis (largely associated with underlying neutropenia and/or macrophage dysfunction), APBA pathophysiology stems from immune deviation toward florid Th2 responses and a component of eosinophilic inflammation, suggesting different immunopathogenic mechanisms. Arising from *A. fumigatus* activation of PRRs and proteolytic activity, epithelial, and dendritic cells drive Group 2 innate lymphoid cells (ILC2) and Th2 differentiation [22]. 

Emerging research is beginning to elucidate the mechanisms that shift the T-helper cell response to *A. fumigatus* away from Th1, in favor of a Th2 response. Bhushan *et al.* investigated *Aspergillus* signaling pathways in human bronchial epithelium and found that *Aspergillus* inhibited interferon (IFN)-β signaling through the JAK-STAT1 pathway which reduced the chemokine CXCL10, thus skewing epithelial responses away from Th1 and towards Th2 [23]. Homma *et al.* built on this work, elucidating how *A. fumigatus* inhibits the IFN signaling pathway. They found that exposure of bronchial epithelial cells to *A. fumigatus* activated protease-activated receptor-2 and tyrosine-protein phosphate nonreceptor type 11 which in turn suppressed CXCL 10 [24]. Intuitively, this data from lung epithelial studies is a persuasive model for studying human responses to inhaled allergens. 

Becker *et al.* employed peripheral mononuclear cells (PBMCs) to investigate cytokines and PRRs important for the Th2 response to *Aspergillus*. They reported that *Aspergillus* conidia play a primary role in the induction of a Th2 response in human PBMCs. Rather than the previously-described PRRs (dectin-1 and TLR2) identified in invasive aspergillosis models, Becker *et al.* suggest that PAMPs present in the *Aspergillus* conidial cell wall act through a complement receptor 3 (CR3) PRR pathway in PBMCs, which leads to phagocytosis of the conidia, and ultimately a Th2 response [25]. The pathophysiologic relevance of this finding generated in PBMCs remains to be determined. 

**Figure 1:**
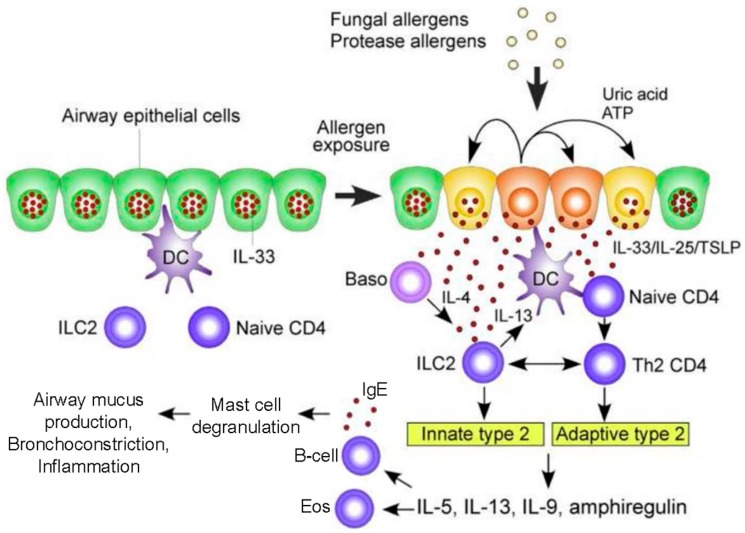
A hypothetical working model to describe the roles of molecular and cellular elements of the local innate response to *Aspergillus* in driving T helper type 2 adaptive immunity in the airway exposed to fungal allergens. In the resting condition, IL-33 is stored in the nucleus of airway epithelial cells. Exposure to fungal allergens (prominently including fungal proteases) induce the extracellular release of IL-33 and production of IL-25 and TSLP by the airway epithelium. Autocrine secretion of ATP and uric acid likely play a role in regulating epithelial release of IL-33. IL-33, IL-25, and TSLP activate innate lymphoid type 2 cells (ILC2) to produce a large quantity of type 2 cytokines including IL-5, IL-13, IL-9, and amphiregulin. Type 2 cytokines drive differentiation of B cells to secrete IgE (thereby sensitizing mast cells and basophils as allergic effectors) and attracting and activating eosinophils. IL-33, IL-25, and TSLP also act upon dendritic and naïve T cells to drive CD4+ T cell differentiation to a Th2 response. Basophil-derived IL-4 may facilitate ILC2 production of cytokines. ILC2-derived IL-13 enhances antigen uptake and migration of dendritic cells and promotes proliferation and differentiation of Th2-type CD4^+^ T cells. In addition, ILC2s and Th2-type CD4^+^ T cells may interact directly to sustain production of type 2 cytokines. Abbreviations: Baso, basophils; DC, dendritic cells; TSLP, thymic stromal lymphopoietin; ATP, adenosine triphosphate; IL, interleukin. Modified with permission from [22].

Epithelial and dendritic cells drive Th2 differentiation via Th2 polarizing chemokines (CCL17) and cytokines (IL-4, IL-5, IL-9, IL-13, IL-25, IL-33, Thymic stromal lymphopoietin (TSLP)). In addition, CCL17 activates regulatory T-cells to suppress Th1 and macrophage responses. Critically, unlike Th1 activation, the Th2 response does not eliminate *A. fumigatus* [26]. Rather, in response to *A. fumigatus* there is a marked acute, but persisting, inflammatory airway response associated with CXCR4+ granulocytes, with significant neutrophilia and eosinophilia [27]. Activated Th2 cells release cytokines (IL-4, IL-5, IL-13) that lead to eosinophil activation as well as B cell differentiation. The resultant IgE antibodies trigger mast cell and basophil degranulation upon exposure to *A. fumigatus* allergens [17,28]. Innate ILC2 cells and adaptive CD4+ Th2 cells release type 2 cytokines (e.g., IL-5, IL-9, IL-13, amphiregulin) which activate mast cells and eosinophils (Figure 1) [29]. The role of Il-17 continues to be defined, though it appears to contribute to the recruitment of neutrophils and airway inflammation [26]. Ultimately, this robust Th2 inflammatory response is deleterious, resulting in the airway mucus production, hyper-responsiveness, inflammation, and bronchiectasis that characterize ABPA [30].

### 1.2. Diagnosis of ABPA

ABPA was first described in 1952 when Hinson, Moon, and Plummer described three patients with recurrent wheezing, pulmonary infiltrates, eosinophilia in blood and sputum, and brown plugs or flecks in expectorated mucus. Clinically, it presents as increasingly severe asthma, or cystic fibrosis exacerbations. There are no specific clinical or physical examination findings to ABPA. Symptoms can range from recurrent pulmonary exacerbations with cough, wheeze, and shortness of breath to systemic features with fever, anorexia, and malaise. Physical examination findings can range from a normal examination to digital clubbing, auscultatory fine crackles, or bronchial breath sounds. Immunologically, ABPA is characterized by local and circulating IgE and IgG *Aspergillus*-specific antibodies, immediate skin test reactivity to *Aspergillus,* local and peripheral eosinophilia, increased IL-2 receptor levels, and raised total serum IgE levels. Radiographically, it includes new lung infiltrates or bronchiectasis on chest imaging. Pathologically, ABPA is characterized by one or more of the following: mucoid impaction of bronchi, bronchocentric granulomatosis, eosinophilic pneumonia, and exudative or obliterative bronchiolitis. 

However, it was not until 1977 that Rosenberg, Patterson, and colleagues in Chicago proposed a set of diagnostic criteria (Table 1) [31]. If a patient had six of the seven proposed major criteria, then a diagnosis of ABPA was considered likely, while the presence of the seventh, proximal bronchiectasis, made the diagnosis certain. 

Since then, as laboratory and clinical medicine have continued to advance, diagnostic criteria for ABPA have been modified, especially in light of improved and more specific serologic and radiographic testing (Table 2) [32]. However the diagnosis of ABPA still remains somewhat nebulous, as it is a result of satisfying a set of particular, inherently non-specific, criteria, rather than a single pathognomonic serologic, clinical, or radiographic characteristic exhibiting high sensitivity, specificity, positive and negative predictive values [33]. Additionally, current guidelines for a diagnosis of ABPA in cystic fibrosis must take into context overlapping aspects of CF lung disease *per se*, including bronchiectasis, transient infiltrates, and partially-reversible obstructive pulmonary physiology and symptoms, and ABPA, resulting in modest modifications of the Rosenberg-Patterson criteria (Table 3) [34]. The critical diagnostic elements of asthmatic ABPA have generally been adopted, so long as the CF patient exhibits a suggestive “asthmatic” component of lung disease (hyper-reactivity or reversibility on pulmonary-function testing and/or wheezing that is responsive to bronchodilators or corticosteroids). 

With the recognition of a spectrum of allergic fungal airway disease ranging from simple sensitization to fungal asthma to severe asthma with fungal sensitization to ABPA, we are discovering that the diagnosis of APBA lies along a continuum, with a gradation of symptoms, and serologic and radiographic features [35]. There is also a potential lack of clarity after the initial diagnosis, which has led to the concept of staging or classification to take into account the dynamic nature of many of the findings associated with a diagnosis of ABPA [32].

An ongoing and significant source of uncertainty in ABPA diagnosis is the lack of standardization of several laboratory tests and/or values. Of the current laboratory tests that are employed to make the diagnosis, only a few, such as total and *Aspergillus*-specifc serum IgE and peripheral eosinophil counts, have the ability to be standardized and automated. Even for these, the problem of cutoff values from a normal range in differing populations is not solved [32,33,34,36]. Other serologic markers, such as precipitins and IgG antibodies to *Aspergillus,* are currently not standardized and a variety of methods of differing sensitivity, specificity, and cutoffs are employed [37,38]. Finally, although recommended diagnostic criteria allow the use of either skin testing or an *in vitro* *Aspergillus* IgE antibody assay, these evaluations are not always congruent in result [39].

There has, thus, been substantial interest in new and less subjective forms of testing that may indicate fungal sensitization and a host inflammatory response to fungal pathogens. For example, some specific recombinant *Aspergillus* antigens, such as *Asp f3* and *Asp f4*, are expressed only in hyphae and, therefore, may differentiate simple sensitization from more serious allergic *Aspergillus* phenotypes including ABPA. In general, use of recombinant *Aspergillus* allergens in specific IgE assays has been more promising for asthma than CF populations; moreover, such assays are not widely available [40]. Other proposed biomarkers, such as serum thymus activated- and regulated chemokine (TARC/CCL17), have been reported but require validation [41].

There are several characteristic radiographic abnormalities that have been associated with ABPA, some of which are of more recent recognition [42]. Most commonly seen is a large homogenous shadow in one of the upper lobes, without an associated volumetric change. These pulmonary infiltrates will often correlate with clinical symptoms; however, in numerous cases, infiltrates are present in asymptomatic patients, or completely absent in symptomatic ones. Findings such as infiltrates, consolidation, and mucoid impaction can be transient; others, such as bronchiectasis or fibrosis, are more persistent or permanent. Chest high-resolution CT scans in ABPA most commonly show central bronchiectasis, with upper lobe predominance and bronchial wall thickening. While some patients may not have bronchiectasis, its presence, especially multi-lobar central or proximal bronchiectasis on high-resolution CT (HRCT) scan warrants further evaluation for ABPA. The active inflammatory component of ABPA clinically manifests as excessive mucus secretion, which translates into mucoid impaction seen on chest CT. The closest to a pathognomonic radiographic finding is the occurrence of hyper-attenuating mucoid impaction on HRCT, but it is only found in ~20% of ABPA patients on presentation [43]. This HRCT finding has also been shown to correlate with immunological severity and propensity for relapse [44]. It is important to note that a normal radiographic appearance does not completely exclude the diagnosis of ABPA. In addition to those individuals who fulfill clinical, radiographic, and laboratory criteria for a diagnosis of ABPA, there are a subgroup of patients who have clinical symptoms and positive serologies suggestive of ABPA, but who lack radiographic evidence of bronchiectasis, possibly representing a prodromal phase; this constellation has been termed ABPA-serologic (ABPA-S) [32,45]. This category remains an area of uncertain clinical importance, as it is unclear as to whether or not these individuals represent an inherently less aggressive form of ABPA, or an earlier “prodromal” stage that has not yet progressed to overt structural lung damage. There is some evidence that the latter may be the case in at least some individuals [46].

Another test that has been examined secondary to the need for a simplified, yet robust, diagnostic tool is the basophil activation test (BAT), which can be performed on whole blood measuring histamine release or specific cellular activation markers using flow cytometry. Basophils are considered an integral feature of allergic responses exhibiting functional aspects of both innate and adaptive immunity. The flow cytometric BAT measures basophil activation by detecting upregulation of certain surface activation markers such as CD203c upon stimulation with allergen to which a patient is already sensitized. It has been shown that the BAT can reliably and stably discriminate between colonization with and sensitization to *Aspergillus* in patients with cystic fibrosis [46,47]. Furthermore, when used in combination with increased *Aspergillus* specific serum IgE levels and total IgE levels, it correctly identified all cases of CF with ABPA that met consensus criteria [48]. While not rigorously studied, these results may also prove true for patients with asthma [46].

Overall it is imperative that we establish objective, testable, consistent criteria that include radiographic, laboratory, and clinical findings to help better diagnose, classify, and create a standardized patient cohort who will benefit most from aggressive and timely therapies. Once this is established, it will be easier to advocate for the routine testing of ABPA as a source of difficult asthma, or recurrent pulmonary exacerbations of cystic fibrosis.

## 2. Treatment of ABPA

The overall goals of treatment of ABPA include reduction of symptoms of either asthma or CF, reducing pulmonary inflammation, and treatment of exacerbations of ABPA to prevent progression to end-stage fibrotic lung disease [49]. Treatment of ABPA in asthma is the essentially the same as the treatment of ABPA in CF. There is an added difficulty with CF patients as the diagnosis of ABPA is challenging as some of the diagnostic criteria of ABPA overlap with common manifestations of CF. Both CF and ABPA cause similar clinic and radiographic symptomatology as to make distinction between the two diseases difficult [34]. Additionally, CF patients also have increased susceptibility to side effects and toxicities of treatment due to their underlying condition. In both asthma and CF, early diagnosis and treatment can prevent progression to end-stage fibrotic lung disease.

### 2.1. Glucocorticosteroids 

*Oral Glucocorticosteroids*. For over 35 years, systemic glucocorticosteroids have been the mainstay of treatment of ABPA [50]. Systemic steroids have been shown to be an effective first line treatment for APBA in both asthma and CF. This is based on early uncontrolled studies that showed significant improvement after the initiation of steroids which combat the inflammatory response in response to the antigens of *A. fumigatus* [51,52]. Although the general efficacy of systemic steroids is agreed upon, the optimal dosing and duration of treatment is not clearly defined. The most referenced regimen for initial treatment of ABPA (with new pulmonary infiltrates) has been with oral prednisone 0.5 mg/kg daily for 1–2 weeks, then on alternate days for 6–8 weeks, then followed by a slow taper by 5–10 mg every two weeks [52,53,54]. Agarwal *et al.* described a more aggressive approach with treatment dose of 0.75 mg/kg/day for six weeks, then 0.5 mg/kg/day for six weeks, followed by a tapering dose of 5 mg every six weeks for a total duration of 6–12 months [55]. Recently, Agarwal *et al.* performed a randomized controlled trial in patients with asthma and ABPA comparing the efficacy and safety of the two regimens [56]. The 0.5 mg/kg/day regimen was referred to as the “medium dose”, while the 0.75 mg/kg/day regimen was referred to as a “high dose” regimen. Previous studies had looked at each regimen individually and there was some suggestion that high dose would be superior in prevention of exacerbations [55]. This study was the first randomized controlled trial to compare two steroid regimens and determined that the medium dose oral glucocorticosteroids (prednisolone) was effective and safer than the high dose in treatment of ABPA [56]. The number of subjects with exacerbation after one year and glucocorticoid-dependent ABPA after two years was the same in both groups. Secondary outcomes showed that patients receiving high dose steroids were more likely to show a response after six weeks of treatment and show a decline in IgE serum levels. However, improvement in spirometric parameters at six weeks and time to first exacerbation (since stopping steroids) were similar in the two groups. As expected, side effects were more frequently seen in the high dose group as the cumulative dose of glucocorticoid was much higher. Side effects often seen with prolonged use of steroids include hyperglycemia, Cushing’s disease, weight gain, and osteoporosis [57]. Of note, all of these patients were on inhaled corticosteroids and long-acting beta agonists as well. This study is important as it directly compared steroid dosing; however, the study only enrolled patients with asthma complicated by ABPA [56]; therefore, the results may not be applicable to patients with cystic fibrosis. 

Response to treatment should be monitored using multiple modalities. Treatment is monitored clinically by assessing change in symptoms associated with exacerbation, including fever, wheezing, dyspnea, and chest pain. Additionally, pulmonary function testing offers objective data in assessing patient lung volumes, flows and diffusion capacity. Changes in these parameters could indicate an exacerbation. Diagnostic imaging with high resolution CT or chest roentgenogram should be repeated after 4–8 weeks of treatment to assess persistence or clearance of lung infiltrates [53,54]. Total serum IgE level is another useful marker to guide treatment and should be checked every 6–8 weeks after start of glucocorticosteroid therapy and every eight weeks for at least one year [53]. While the elevated IgE levels are not expected to return to normal, despite treatment, it is useful to compare follow up values to each patient’s unique baseline level. In a recent study increases in the total IgE serum level of >50% accompanied exacerbations in >90% of patients [58].

*Intravenous Glucocorticosteroids***.** There is continued exploration of the role of intravenous glucocorticosteroids, specifically “pulse” steroid therapy. Pulse steroid therapy consists of Intravenous methylprednisolone (10–20 mg/kg/day) infused daily for three consecutive days every four weeks. In the literature, there are no controlled trials comparing oral steroids to intravenous steroids. There are several cases reported where IV methylprednisolone was given to steroid-dependent ABPA CF patients who either were not well controlled on conventional therapies (oral prednisolone and oral azole antifungal) or had significant side effects to the oral glucocorticosteroids [59,60,61,62]. A single report presented two adults with severe steroid-dependent asthma complicated by ABPA who had clinical improvement after initiation of pulse steroid therapy [59]. In most cases, pulse IV steroid therapy was well tolerated and patients showed clinical improvement with minimal side effects. They achieved enough disease control to allow discontinuation of the pulse therapy after 6–12 months [59,60,61]. However, these reports had few patients and were not controlled studies. There is no long-term follow up data to further explore the effect this treatment plan had on clinical outcomes. 

*Inhaled Glucocorticosteroids.* Inhaled corticosteroids achieve high concentrations in the tracheobronchial tree with minimal systemic effects and have a clear added benefit in the treatment of underlying asthma [32]. However, for decades, researchers have failed to define a role of inhaled corticosteroids in the treatment of ABPA and their precise place remains elusive. A double-blind placebo controlled trial of beclomethasone did not show any clinically significant improvement [63]. A small case series suggested that inhaled corticosteroids may be a useful adjunct in treatment of ABPA [64]. One study looked specifically at serologic ABPA in asthma, using high-dose inhaled corticosteroids in combination with a long-acting beta agonist [57]. While there was some improvement in asthma symptoms, total serum IgE increased and patients improved only after receiving oral steroids, leading the investigators to conclude that there is no role for inhaled corticosteroid monotherapy in the treatment of serologic ABPA. 

### 2.2. Antifungal Agents

As described above, systemic glucocorticosteroids are the mainstay of treatment and the most effective in treatment for the acute phase of ABPA; however, chronic use with is associated with increasing risk for toxicity and side effects. Adding an antifungal agent to the regimen may have a steroid-sparing effect, reducing the need for steroids to control inflammation [65]. Early descriptions of treatment of ABPA with antifungals go back to 1967, as reported by Stark [66]. Antifungal agents with activity against *A. fumigatus* are recommended as an adjuvant or second line therapy in ABPA for both CF and asthma [32,34]. Azole antifungal agents work by inhibiting ergosterol synthesis in the fungal cell membrane and thereby inhibit fungal growth [67]. Azoles are used to reduce the antigen burden arising from fungal colonization of the airway. It is then expected that the reduction in antigenic stimulation would result in decreased inflammation and reduced disease severity and progression [65]. 

Much of the literature regarding effectiveness of antifungal therapy is limited to a small number of controlled trials and several case reports and case series with heterogeneous patient populations and treatment regimens. In exploring the role of oral antifungals, there are three randomized, double-blind, placebo-controlled trials that included asthmatic patients with ABPA [68,69,70], but no controlled trials in cystic fibrosis ABPA. 

*Ketoconazole.* An early, small open label study in 1984 by Fournier, did not demonstrate any benefit from ketoconazole; although another small study with patients with mild disease did show some improvement in biomarkers, including specific IgG antibody to *A. fumigatus* and total and specific IgE [67]. A randomized controlled trial by Shale *et al*. in 1987 showed improvement in subjective symptoms and decreased biomarkers; although no objective improvement in lung function [68]. However, given the adverse events risk associated with chronic use of ketoconazole, researchers recommended consideration only in the face of serious disease that could lead to either disability or death. In general, this has been the standard practice as ketoconazole is not typically used for long-term therapy given the increased incidence of toxicity compared to other azoles. Specifically, there is an increased risk of adrenal dysfunction and hepatic disease [65,67].

*Itraconazole.* Itraconazole is an orally-administered triazole that has fewer side effects and a wider spectrum of activity compared to ketoconazole. There have been open-label case series that suggest benefit in the treatment of ABPA in patients with and without cystic fibrosis [65]. There are two randomized controlled trials using itraconazole in ABPA. Stevens *et al.* in 2000 published findings from their randomized, double-blind trial of treatment with either 200 mg of itraconazole twice daily or placebo for 16 weeks in patients [69]. These patients met immunologic and pulmonary function test criteria for corticosteroid-dependent ABPA. A response was defined as at least a 50% reduction in steroid dose, a 25% reduction in serum IgE concentration and either an improvement of 25% in exercise tolerance testing or pulmonary function testing or a resolution of pulmonary infiltrates on imaging. There was also a follow-on open-label arm of the trial where all patients received itraconazole 200 mg daily (a lower dose than in the placebo-controlled trial) for 16 weeks. The study demonstrated that in patients with corticosteroid-dependent ABPA adding itraconazole can lead to clinical improvement without significant risk of toxicity. Additionally, the lower dose used in the open label trial showed benefit as well [69]. A limitation of the study is that there were no patients with CF, limiting the generalizability of the results. Wark *et al.* conducted a randomized, double-blind, placebo-controlled trial in 29 patients with asthma and stable ABPA [70]. The study demonstrated a decrease in sputum eosinophils, serum IgE levels and *A. fumigatus-*specific IgG levels. Clinically-active azole therapy also reduced the incidence of exacerbations [70]. These findings are consistent with what has been found in other retrospective case reports and open-label studies [65,71,72].

*Voriconazole and posaconazole.* While itraconazole has been shown to be an effective option, there are patients who continue to have symptoms despite treatment, or who develop severe enough adverse effects to stop treatment. Newer triazoles, such as voriconazole and posaconazole, offer similar antifungal coverage, but have better bioavailibilty and have been better tolerated in some patients. A retrospective study by Chishimba *et al.* looked at 25 asthmatic patients with ABPA or severe asthma with fungal sensitization treated with voriconazole or posaconazole [73]. All of the patients had failed a course of itraconazole or developed adverse events while on itraconazole and stopped treatment. There were 33 courses of therapy analyzed (24 voriconazole and nine posaconazole). Patients in both treatment groups demonstrated a marked reduction in use of oral corticosteroids as well as a reduced need for rescue dosing of a short-acting beta agonist. They also reported improved overall health status. This small study suggests that both voriconazole and posaconazole can be alternative antifungal therapy in patients with ABPA; however, it is limited as a retrospective study in an asthmatic population.

*Special considerations with azoles*. Itraconazole is a weak base, requiring an acidic environment to be most effective. It is also lipophilic and distributes widely to the respiratory track with 95% bound to albumin. This is important when treating patients who are malnourished, as they may have increased tissue deposition in the setting of hypoalbuminemia. Additionally, clinicians should be careful in using azoles concomitantly with a number of systemic or inhaled glucocorticosteroids. Studies have shown that azoles can increase the serum concentration of methylprednisolone by slowing its metabolism via inhibition of hepatic CYP3A [74]. Reports have also shown that use of itraconazole with inhaled corticosteroids has been associated with increased incidence of adrenal insufficiency [67]. The effects are typically attributed to the interaction of azoles with drugs that are metabolized by cytochrome P450 enzymes, because azoles are competitive inhibitors of CYP 3A4, 2C9, and 2C19 [34,65]. This likely explains the other adverse events seen with use of azoles, including nausea, vomiting, and hepatotoxicity [65]. The most common adverse effects associated with voriconazole include vision disturbances, transaminase elevations, nausea, vomiting, diarrhea, central nervous system abnormalities, and skin toxicities [75,76,77,78]. Voriconazole is also associated with photosensitivity and photodamage. Chronic use (>12 months) is associated with cutaneous malignancy, specifically cutaneous squamous cell carcinoma (C-SCC) [79]. Special caution is recommended in lung transplant recipients who are already at increased risk for C-SCC [80]. Patients with cystic fibrosis who take voriconazole may be at increased risk for cutaneous complications. Cystic fibrosis patients typically receive vitamin A (as well as other fat-soluble vitamins) supplementation for pancreatic insufficiency. Voriconazole inhibits hepatic enzyme activity needed to metabolize all-trans retinoic acid, a known metabolite of retinol (vitamin A). Trans retinoic acid is phototoxic in high concentrations and, by extension, is believed to contribute to the increased occurrence of phototoxicity among CF patients on voriconazole [75].

There are several medications commonly prescribed for patients with cystic fibrosis and asthma that have known interactions with azoles, including omeprazole, ibuprofen, calcium channel blockers, and the new cystic fibrosis transmembrane conductance regulator (CFTR) modulator drugs ivacaftor and lumacaftor. Therefore, the initiation of azole therapy for ABPA in CF should be weighed against these potential drug-drug interactions. A recent paper by Harrison *et al.* suggested that using Itraconazole with ivacaftor may actually take advantage of this drug-drug interaction by improving the efficacy of the CFTR modulator and allowing ivacaftor dose reduction, an interesting consideration in light of ivacaftor’s >$300,000 annual per patient cost [81].

Therapeutic drug monitoring is important in all patients, but especially in patients with cystic fibrosis, as the pharmacokinetics in these patients can vary significantly. Previous studies have shown that in people with cystic fibrosis, itraconazole absorption can be poor and unreliable [82]. A study by Berge *et al.* suggested difficulty in achieving appropriate drug levels in CF patients after lung transplant [83]. However, this may not be relevant to pre-transplant patients with CF as Clifton *et al.* reported that oral voriconazole is rapidly absorbed in patients with CF with a peak concentration 1–2 h following ingestion [84].

Also complicating the management of ABPA is the emergence of azole-resistance in *A. fumigatus* [85]. The emergence of resistance in *A. fumigatus* suggests the need for *in vitro* susceptibility testing. To try and prevent azole resistance in an individual patient it has been suggested that therapeutic drug monitoring be used to ensure against suboptimal treatment, which could increase resistance.

*Inhaled amphotericin B.* In an attempt to avoid the potential adverse effects of oral azoles there has been consideration of inhaled antifungal therapy. Amphotericin B was first isolated in 1956 and is the broadest-spectrum antifungal available [86]. Its general use is limited in part due to the various toxicities associated with intravenous use. Acute side effects include fever, chills, anaphylaxis, cardiac arrhythmia, and liver failure, with long term effects, such as renal tubular acidosis and interstitial nephritis. Amphotericin is less toxic when incorporated into a liposomal bilayer and inhaled amphotericin B offers the advantage of allowing an adequate minimal inhibitory concentration for *A. fumigatus* to be achieved relatively easily with minimal systemic distribution and side effects [86,87]. Until recently there had not been any randomized controlled trials assessing the efficacy of inhaled amphotericin B in the treatment of ABPA. There have been case series reporting efficacy in both asthma ABPA and cystic fibrosis ABPA. Proesmans *et al.* described the treatment course of seven patients with cystic fibrosis with recalcitrant ABPA. For six of the seven patients, inhaled amphotericin B allowed steroids to be discontinued and these patients remained free of relapse for years [88]. Recently, Ram *et al.* reported results of a randomized controlled pilot trial of nebulized amphotericin for maintenance of remission in patients with asthmatic ABPA [89]. The study involved 21 subjects who received either nebulized amphotericin B plus nebulized budesonide *versus* nebulized budesonide alone. The primary outcome was the time to first exacerbation with secondary outcomes including the number of exacerbations, lung function, serum IgE levels and asthma control test scores. Although the study was small it found that nebulized amphotericin B was beneficial in decreasing the frequency of exacerbations in patients with asthmatic ABPA. A randomized controlled trial in patients with cystic fibrosis ABPA has not been done, but case reports suggest a possible benefit to this patient population [88,90].

While more study is warranted, findings to date suggest that for patients with recurrent exacerbations, treatment with an antifungal should be considered. Most of the focus of discussion regarding antifungals has been on using them as an adjuvant to systemic glucocorticosteroid therapy. It is unclear as to whether antifungals can be an effective first line treatment for patients with ABPA. A randomized control trial comparing monotherapy of itraconazole *versus* prednisolone in ABPA is underway (Clinicaltrials.gov identifier NCT01321827: https://clinicaltrials.gov/) and will hopefully shed more light as to whether antifungal monotherapy is a reasonable option.

### 2.3. Immunotherapy

*Omalizumab.* Omalizumab is a humanized monoclonal antibody to IgE that binds to free serum IgE, interfering with IgE binding to its high-affinity receptor on mast cells and basophils, and also downregulating IgE receptors on lymphoid cells. It has been shown to be effective in the treatment of poorly-controlled severe allergic asthma in adults and children who require continuous or frequent treatment with oral corticosteroids. Several studies have shown that treatment with omalizumab is associated with increased lung function (increased FEV_1_), decreased respiratory symptoms and decreased need for systemic steroids in patients with allergic asthma [91,92]. The most recent Cochrane review determined that use of omalizumab is associated with reduced exacerbations and reduced hospitalization rates in adults and children with allergic asthma [93]. 

The dosing recommendations for omalizumab are currently based on treatment of asthma with the goal of decreasing the total free IgE to less than 50 ng/mL (20.8 IU/mL). The drug is delivered either once every two or four weeks by subcutaneous injection. The package insert dosing is based on patient weight and total IgE level, to a maximum dose of 750 mg monthly; the pre-treatment total IgE serum upper level limit is listed at 700 IU/mL. Patients with ABPA usually exceed the current dose parameters due to their extremely high serum IgE levels. However, in the past eight years there have been many reports and open-label trials in >100 published cases that report benefits of usual or only modestly increased omalizumab doses in patients with ABPA, suggesting that it may be an effective steroid-sparing treatment that reduces exacerbations of ABPA in patients with asthma or cystic fibrosis [28,94,95,96,97,98,99,100,101,102]. In addition, the general validity of monitoring total serum IgE levels with standardized commercially available assays in response to omalizumab therapy in ABPA as well as asthma has been established [103]. Until recently, however, lack of a placebo-controlled trial has restrained a recommendation on omalizumab use in ABPA [104]. Voskamp *et al.* have now reported the first randomized, blinded, controlled trial with 13 asthmatic ABPA patients who participated in a four-month treatment with omalizumab (750 mg monthly) or placebo, followed by a three-month washout period in a cross-over design [105]. The study met its primary endpoint of showing a significant reduction in exacerbations among patients receiving active treatment with omalizumab. It also met secondary outcomes of decreased mean fractionated exhaled nitric oxide, a known marker of lower airway inflammation, and reduced basophil reactivity, a measure of allergic cellular response, during treatment. Patients in the study were on oral steroids; however, investigators did not comment on steroid dosing during or after treatment. Previous uncontrolled studies strongly suggest a reduction or discontinuation in oral steroid dosing in patients treated with omalizumab is often achieved. The Voskamp study confirms the safety and effectiveness of omalizumab in the treatment of ABPA, despite high IgE levels, but does not resolve the relative roles of steroids, antifungals, and immunomodulation in the overall management of ABPA. 

There is still a need for a controlled trial of omalizumab for cystic fibrosis ABPA, as these patients were excluded from the study of Voskamp *et al.* [105]. There has been an attempt at a multi-center, industry-sponsored randomized double-blind, placebo-controlled, trial for patients with cystic fibrosis ABPA. In this study, participants received omalizumab 600 mg or placebo injections daily for six months, while receiving itraconazole and oral steroids. This study was prematurely terminated due to participant drop out, likely due to intolerance of the arduous regimen (Clinicaltrials.gov identifier NCT00787917).

ABPA is a challenging, complex chronic disease. Treatment with intravenous pulse glucocorticosteroids, oral or inhaled antifungals, and anti-IgE, have been shown to be safe and effective adjuncts to the mainstay of therapy, oral glucocorticosteroids. While there have been more controlled studies over the past few years, there is still a lack of controlled studies testing the effectiveness of pulse steroids, antifungals and anti-IgE particularly in patients with cystic fibrosis ABPA. Additional adjunctive therapies suggested in the literature include nebulized hypertonic saline to reduce sputum viscosity and aid in expectoration of mucus plugs [106], as well as chronic use of azithromycin for its anti-inflammatory effects on the airways [107,108]. While diagnostic bronchoscopy has been suggested in cases lacking conventional criteria [109], therapeutic bronchoscopy has also been considered, and a paper by Khalil described the use of bronchoscopy with therapeutic bronchoalveolar lavage in 78 ABPA patients to remove mucus plugs in patients with high attenuation mucus or central bronchiectasis; but while there was a significant reduction in IgE (all patients were also treated with prednisolone and itraconazole) there was no change in the clinical recurrence rate as compared to ABPA patients not undergoing bronchoscopy [110]. New treatment modalities being investigated include further immune response modulation, reducing the inflammation caused by sensitization to *A. fumigatus* or other fungi. For example, studies have shown that *A. fumigatus* produces a metabolite, gliotoxin, that downregulates the vitamin D receptor on macrophages and airway epithelial cells. This downregulation leads to increased production of Th2 cytokines IL-5 and IL-13 [111]. These findings suggest antifungal treatment to reduce fungal burden, used in concert with vitamin D supplementation, may offer benefit [111,112]. Additionally, targeted anti-Th2 therapies such as monoclonal antibodies directed against Th2 cytokines or their cognate receptors that are in clinical trials for severe asthma may be applicable to ABPA [113]. Finally, mitigating environmental exposure to airborne *Aspergillus* conidia is also important in patients with ABPA and fungal sensitization in general. Patients should consider avoiding gardening, farm activities, and exposure to building renovations, or moldy/dusty environments that may contain high amounts of *Aspergillus* conidia. 

## 3. Conclusions

Our understanding of the pathogenesis of ABPA is still evolving; in particular, the early determinants of immune deviation to a Th2 profile in immunocompetent individuals are not yet fully elucidated, although progress using *in vitro* systems and animal models is occurring. Many obstacles to rapid and accurate diagnosis remain, with regard to both standardization of existing laboratory tests and development of new algorithms. In this regard, combination of serological testing of total and *Aspergillus*-specific IgE, in concert with the flow cytometric basophil activation assay, appears promising. Finally, in determining which treatment should be used with each patient, the goal should be to offer maximum benefit to each patient with the least occurrence of adverse reactions and toxicity. Some patients may require a combination of several different therapeutic modalities before symptoms are kept under reliable control. While on treatment, patients should be initially followed every 6–8 weeks, checking serum IgE levels, chest radiographs or CT, spirometric lung function tests, and report of symptoms and quality of life. Improvement in symptoms may offer an opportunity to wean or stop certain medications associated with adverse events.

With proper treatment ABPA is a controllable, albeit chronic, illness. Response to treatment in ABPA needs to be evaluated in multiple domains over time, including clinical (symptoms, exacerbations), immunologic (total IgE), physiologic (spirometry), and structural (chest HRCT or radiograph) evaluations and measures [32,50,51,52,53]. While data on long-term prognosis is quite limited, treatment is effective in maintaining lung function and overall health [114,115], but late diagnosis and/or untreated ABPA leads to progressive and potentially fatal pulmonary fibrosis [116,117].

For current and developing therapies, well-designed controlled trials are needed, especially for the cystic fibrosis ABPA population. Continued studies, ideally with multi-center collaboration, will help to further refine treatment of this complex disease in a way that increases effectiveness, while optimizing the safety profile and reducing adverse events. 

## Figures and Tables

**Table 1:** Rosenberg-Patterson 1977 criteria for diagnosis of ABPA [31].

Primary criteria (1–6 suggestive, +7 definite)
Episodic bronchial obstruction Peripheral eosinophilia Positive immediate skin test to *Aspergillus* Positive preciptin test to *Aspergillus* Increased total serum IgE History of transient or fixed lung infiltrates Proximal bronchiectasis
**Secondary (supportive) criteria**
Brown plugs/flecks in sputum Positive late (6–12 h/Arthus) skin test to *Aspergillus*

**Table 2:** Modified ISHAM working group 2013 criteria for diagnosis of ABPA [32].

Predisposing asthma or CF Obligatory criteria IgE > 1000 IU/mL and Positive immediate skin test or increased IgE antibody to *Aspergillus* Supportive (≥2) criteria Eosinophila > 500 Precipitins or increased IgG antibody to *Aspergillus* Consistent radiographic opacities

**Table 3:** Consensus Conference 2003 minimal diagnostic criteria for diagnosis of ABPA in cystic fibrosis [34].

Clinical deterioration (e.g., increased cough, wheeze, increased sputum production, decrease in spirometric lung function) Total serum IgE ≥ 500 IU/mL Immediate skin test reactivity or IgE antibodies to *Aspergillus* Precipitins or IgG antibodies to *Apergillus*, OR abnormal or changed chest radiograph or chest HRCT not responsive to antibiotics or physiotherapy
